# Mr. John Tilma

**Published:** 1917-02

**Authors:** 


**Mr. John Tilma,** prominent among Buffalo pharmacists, died suddenly in the building of the Iroquois Gas Co., Jan. 17, aged 62.

